# Temporal shaping of the eastern chants: a case study of the performances of Debussy’s *Prélude Canope*

**DOI:** 10.3389/fpsyg.2026.1855107

**Published:** 2026-07-01

**Authors:** Xuanyun Huang, Yingying Su

**Affiliations:** 1 School of Arts, Sun Yat-sen University, Guangzhou, China; 2 School of Music, Nanjing Normal University, Nanjing, China

**Keywords:** cross-culture comparison, Debussy’s *Préludes*, eastern-inspired style, micro-rhythmic divergence, temporal control, visualization analysis

## Abstract

**Introduction:**

Previous research on Debussy’s *Préludes* has largely focused on harmony and texture, with tempo and rhythm receiving less attention and rarely being supported by quantitative analysis. This study investigates Canope (*Préludes*, *Book II*), a work whose impressionistic, Eastern-inspired soundscape is fundamentally shaped by layered tempo and rubato-driven rhythms.

**Methods:**

Using the visualization platform Vmus, we comparatively analyze 15 recordings (1953–2018) by pianists from French, Russian, Polish, German, and Chinese backgrounds. Macro-level tempo structures and note-to-note inter-onset intervals (IOIs) are examined to capture micro-temporal deviations and phrase-level flexibility, revealing interpretative nuances in rhythmic articulation

**Results:**

Our findings indicate that performances of Canope creatively reconstruct the score through temporal elasticity. A dual pattern emerges across both macro-level shaping and micro-rhythmic rubato: shared characteristics aligning with provisional artistic-tradition clusters, alongside individual idiosyncrasies that resist such categorization. Together, these elements shape the realization of Debussy’s Eastern-inspired imagery.

**Discussion:**

Contemporary trends reveal both cross-school integration and a shift toward personalized artistic expression, with individual deviations acting as sites of innovation and cross-tradition dialogue.

## Introduction

Debussy’s *Préludes* are central to Impressionist musicology, subverting the conventions of traditional Romanticism and striving toward a mode of musical expression marked by heightened freedom, fluidity, nuanced sensitivity, and timbral richness. *Canope,* a compact work from the second book of the *Préludes*, serves as a concentrated exemplar of the composer’s artistic reinterpretation of Eastern aesthetic principles. It achieves this through a fantasized imitation of distant Eastern rhythmic paradigms via the European musical consciousness, employing fragmented rhythmic cells, elastic tempo fluctuations, and the stratification of asymmetrical phrasing.

The importance of rhythm and tempo in Debussy’s music is significant. De Leeuw, (2005) categorize the composer’s rhythmic organization as “floating rhythm,” a 19th-century innovation that challenges metrical conventions to expand temporal–spatial freedom in composition. Despite rhythm’s pivotal role in Debussy’s idiom, analyses of how nuanced temporal control in actual performance shapes the sonic effects of the music remain scarce, and quantitative methodologies are rarer still. Encompassing speed and rhythmic precision, the art of temporal control has long been a central concern for both theorists and performers. Each pedagogical tradition develops its own principles regarding tempo and rhythm, and scholarship has likewise sought to decode their essence. Empirical research in performance studies is primarily structured around two methodological frameworks: stylistic trend studies, which examine historical evolution and stylistic divergence across performers, historical periods, or cultural contexts; and performance modeling, which formalizes stable expressive features to construct conventional performance models.

These frameworks give rise to two distinct analytical approaches to temporal control. Idiographic studies emphasize qualitative analysis of individual performers’ interpretive idiosyncrasies. Performance variation studies focus on comparative analysis of specific musical passages across multiple interpretations. However, both approaches have certain limitations, including a reliance on qualitative description, a narrow sampling scope, and limited generalizability.

In search of a more scientific analytical framework for tempo studies in performance, Zhou and Fabian (2019) constructed a systematic taxonomy for tempo and tempo variation. Their model evaluates individual performer differences across three dimensions: basic tempo, global tempo variation, and local tempo variation, each operationalized as two distinct variables, yielding a total of six variables for precise assessment of stylistic individuality. This three-dimensional model offers an effective and precise methodological framework for comparing performers’ interpretive tendencies, facilitating a comprehensive understanding of individual differences in tempo execution and temporal flexibility. Zhou (2022) later expanded this framework into a detailed treatise.

In examining the shaping and categorization of performance styles, Fabian (2017) employs Deleuzian philosophical concepts such as difference, becoming, and multiplicity to re-examine the evolution of performance styles in J.S. Bach’s solo violin works. Her analysis shows that styles are not static but evolve continuously across generations. Emerging artists of the new generation cultivate “micro-traditions”: context-specific, dynamic performative practices that catalyze stylistic evolution through intercultural innovation. This dynamic evolution frames performance not as the reproduction of fixed norms, but as an active site of stylistic becoming, where tradition is constantly reshaped through individual agency and generational sensibility.

## Methodology

### Research framework

According to the methodological framework proposed by Yang and Huang (2016), visualization analysis in musical performance studies serves as an “augmented listening” tool, a concept initially put forward by Nicholas Cook during his lecture at Nanjing University of the Arts (October 8, 2014). This tool offers an environment to capture subtle details that are hard to accurately perceive through auditory perception alone.

It acts as an integrative intermediary, connecting the synchronic dimensions of musical scores, such as harmonic progressions and formal architecture, with the diachronic parameters of sonic realization, such as tempo inflection and dynamic contour.

Meanwhile, it also synthesizes historically contextualized elements, including score editions, formal structures, artistic conceptions, stylistic conventions, and aesthetic paradigms. It concretizes these abstract dimensions through audible manifestations, visual representations, and quantitative metrics.

Significant applications of this methodological approach include Yang’s (2010) study on Chopin Nocturne and Strauss’s The Blue Danube Yang (2022), Fabian’s (2005) analysis of Bach’s solo violin works, where visualization techniques facilitated a detailed examination of individual recordings, and the collaborative research of Zhou and Fabian (2019), which employed similar methodologies to construct theoretical frameworks for performance analysis.

Following this established methodological guidance, the present study on Debussy’s *Canope* employs recording comparison, visualization analysis (Vmus.net), and IOI analysis. It compares recordings from 15 performers, a relatively limited but representative corpus that subjects each sample to in-depth case analysis. It not only identifies common patterns but also highlights those boundary-defying, uniquely inventive interpretations, ultimately providing a more nuanced and comprehensive understanding of the work’s performance possibilities.

The methodology aims to make each performer’s distinctive artistic treatment more intuitively accessible through visualization techniques, thereby facilitating nuanced discussions of both shared characteristics and individual particularities rather than focusing solely on extracting averages or trend lines.

### Selection of recordings

This study examines recorded performances by 15 pianists (1953–2018) selected according to two criteria: a reputation for exceptional artistry and being representative of a specific artistic tradition (Table 1). These artistic traditions were identified through the pianists’ cultural backgrounds and pedagogical inheritances. We categorized them into six traditions: French, German-Austrian, Italian, Polish, Russian, and Chinese.

**Table 1:** Recording selection of *Canope.*

No.	Grouping	Background	Performer	Year	Label
1	Western Europe	France	Samson Francois	1968	EMI Classics
2	Western Europe	France	Jean-Yves Thibaudet	1996	Decca
3	Western Europe	France	Pascal Roge	2004	ONYX CLASSICS
4	Western Europe	France	Pierre-Laurent Aimard	2012	Deutsche Grammophon
5	Western Europe	Germany/France	Walter Gieseking	1953	Warner Classics
6	Western Europe	Germany	Friedrich Gulda	1969	MPS Records
7	Western Europe	Germany	Claudio Arrau	1979	Philips
8	Western Europe	Italy	Arturo Benedetti Michelangeli	1988	Deutsche Grammophon
9	Western Europe	Italy	Maurizio Pollini	2016	Deutsche Grammophon
10	Eastern Europe	Poland	Krystian Zimerman	1991	Deutsche Grammophon
11	EasternEurope	Russia	Youri Egorov	1983	EMI Classics
12	Eastern Europe	Russia	Sviatoslav Richter	1993	Stradivarius
13	China	China	Fou Ts'ong	1986	Fumao Records
14	China	China	Yin Chengzong	1998	Marco Polo
15	China	China	Chen Sa	2017	ChenSa Studio

Rather than treating these as rigid regional groupings, we adopt “Western European” (French, German-Austrian, Italian), “Eastern European” (Polish, Russian), and “Chinese” as provisional analytical clusters to contextualize the artistic tradition networks. We recognize that these categories reflect scholarly conventions rather than culturally bounded entities.

Critically, while performers from documented artistic traditions often display stylistic tendencies, we treat such correlations as probabilistic inclinations rather than deterministic outcomes. Confounding variables, including individual artistic agency, cross-cultural experiences, and evolving historical contexts, preclude any causal claims. This framework serves strictly as an initial heuristic scaffold. Our primary analytical focus lies in examining how performers navigate tradition through measurable parameters, specifically temporal control, thereby avoiding the reification of cultural categories.

Building on Fabian’s (2017) observation that stylistic synthesis may emerge through *“non-dialectical, non-identity-based modes of thinking”* (Fabian, 2017, para.0.3), our analysis demonstrates how documented artistic tradition networks probabilistically influence certain performance interpretations in this corpus, while individual deviations reveal sites of artistic agency. This reframes artistic tradition not as a deterministic shaper of style, but as one variable among many within a complex system of performative decision-making.

### Technical approach

This study employed Vmus.net, an online visualization analysis platform for music performance research developed by Jian Yang at the Shanghai Conservatory of Music. The platform was initially developed in 2003 and upgraded to a web-based system in 2014. Vmus.net offers multimodal visualizations of audio recordings, including waveforms, spectral analyses, tempo-dynamics curves, Inter-Onset Interval (IOI) deviation plots, and performance worm diagrams. The platform supports online storage and global sharing of analysis results, facilitating remote collaboration and the potential for big data mining.

Compared to Sonic Visualiser, the other music audio analysis application developed at the University of London, Vmus.net shows distinct advantages for tempo and micro-rhythmic analysis. While Sonic Visualiser requires software installation and plugin configuration, with a steeper learning curve, Vmus.net provides immediate web-based access with integrated tempo curve and IOI analysis functions specifically designed for music performance research. By 2016, the platform had attracted over 600 registered users worldwide, and its analysis results were frequently cited in academic publications.

The specific technical approach of this study is as follows:

1. We employ two functions of the Vmus.net platform to obtain visualized graphs of the quantitative data:

Macroscopic Tempo Curve Analysis: Users mark beats during audio playback to generate visualizations that display raw BPM values (red dots), smoothed tempo trends (black line), and an average tempo reference (dashed line). A full-length analysis of the audio of the entire musical work offers a comprehensive understanding of macroscopic tempo structure.

IOI Analysis: It measures the temporal relationships between consecutive notes by marking onset moments within a selected passage. The IOI deviation percentage indicates how much longer (positive) or shorter (negative) the performed intervals are compared to the indicated durations notated in the musical score. The visualization shows deviation percentages vertically against sequential note numbers horizontally, with the IOI duration measured in seconds. This study examines micro-temporal articulation in two structurally significant passages from *Canope* across 15 performances: the expository opening and the incipit of the middle section’s variation sequence, selected for their pivotal roles in formal architecture and expressive character.

2. Upon obtaining the macroscopic tempo structure for each performance, we conducted a comparative analysis that revealed both shared characteristics consistent with our provisional artistic-tradition clusters and individual particularities that elude such grouping. A parallel comparison of the IOI data from two significant passages in each performance yielded the same dual pattern: commonalities aligned with these clusters, alongside distinctive individual particularities that resist categorization.

### Context of the *Prélude*

The term “Canope” originally referred to “Canopus” (known today as Abu Qir), an ancient Egyptian city cited by the Greeks. In French, however, the word also denotes a specific jar-shaped vessel excavated from this city, which was used in the mummification process: the Canopic jar. These jars were used to preserve the internal organs removed from the deceased during embalming, ensuring their availability for the afterlife.

From the Old Kingdom of Egypt until the simplification of funerary rites during the Hellenistic Ptolemaic period, Canopic jars were not only pivotal artifacts in Egyptological archaeology and history but also significant cultural symbols of ancient Egypt.

Eastern culture was a paramount source of inspiration for Debussy’s compositional endeavors. The composer personally attended the Paris Expositions Universelles in 1889 and 1890, where he encountered artistic artifacts and musical performances from East and South Asia. This exposure sparked a profound and enduring fascination within him, fundamentally shaping his subsequent aesthetic trajectory.

The influence of Eastern culture pervades Debussy’s music, manifesting in both formal expressions and aesthetic principles. This influence is particularly direct and explicit in Debussy’s explicitly designated Eastern–themed compositions.

Scott (1998) has argued, *“One might ask if it is necessary to know anything about Eastern musical practices; for the most part, it seems that only a knowledge of Orientalist signifiers is required.”* (Scott, 1998, p. 309).

*Canope* positions its cultural framework within the context of Ancient Egypt. However, its aesthetic discourse lacks the stylistic elements characteristic of Ancient Egyptian music, making the composition unconnected to the authentic musical practices of that period. Instead, it is more of a subjective portrayal of the artifact itself, capturing a reductive exoticism from *“.the city, the temple, the associated rites, the dead civilization itself, and the funeral ideas of a more general nature.”* (Lewin, 1987, p. 66).

The work reflects a fantasized interpretation of Egyptian antiquity, filtered through Debussy’s personal perspective of Eastern imagination. Is it an afterthought of an exhibition? Or a wild fantasy of ancient Egyptian civilization? Thus, it also offers a great deal of room for performance interpretation.

From the perspective of musical narrative, Hoffman R. posits that *Canope* can be interpreted as a complete process of viewing a set of Canopic jars (Hoffman, 2002, p. 107). The narrative arc commences with a slow approach, followed by a meticulous, individual scrutiny of the artifact. As the listener’s imagination is roused by the vessel’s mysterious cultural aura, it gradually transports them back to that bygone era, fostering a spiritual resonance. The experience concludes as these ripples of thoughts gradually fade, marking the end of the contemplative observation.

## Results

### Macroscopic tempo: range

**Figure 1:**
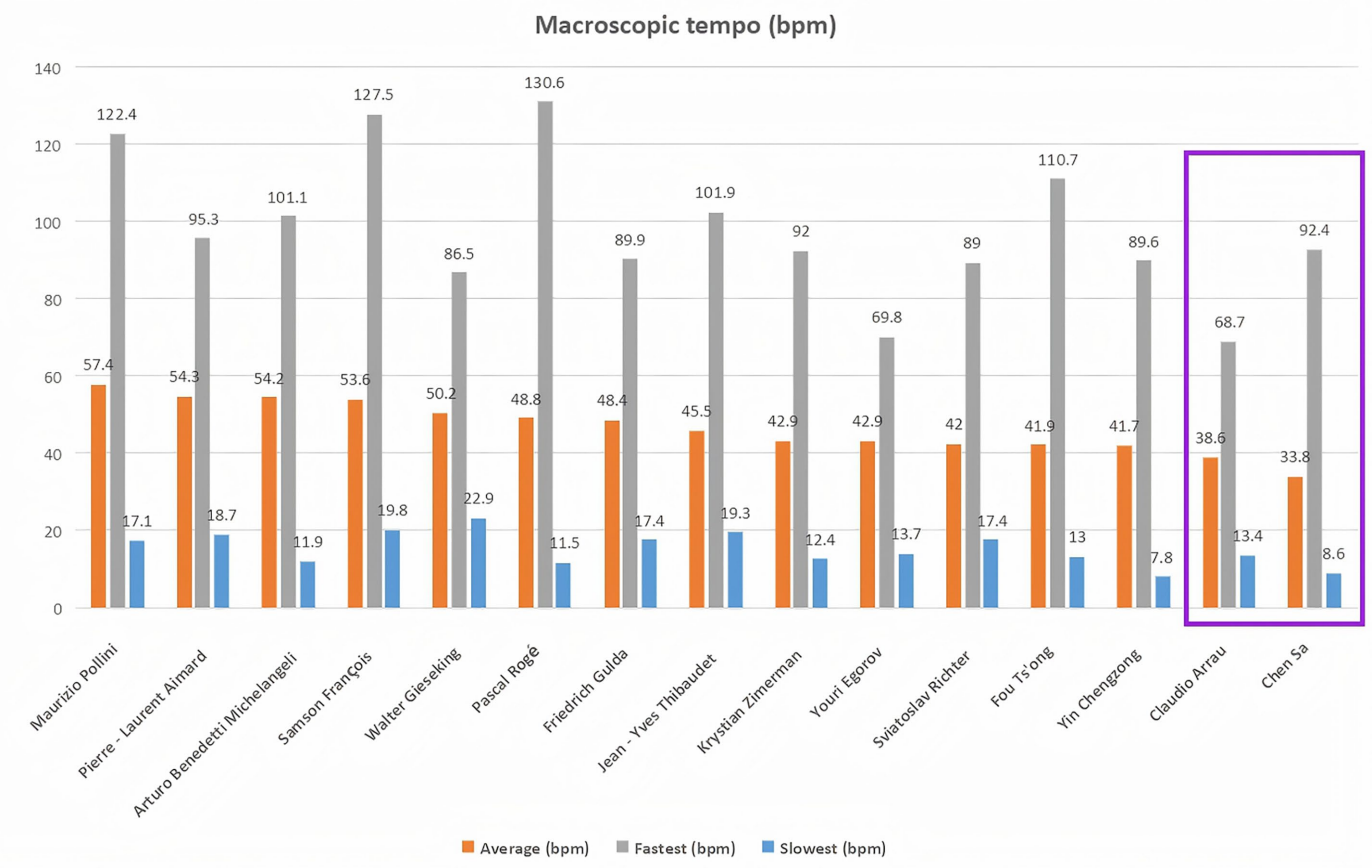
Macroscopic Tempo range.

On a macro level, there is a notable consensus on the treatments of tempo across different recorded versions. The fundamental tempo of the piece is set at a slow pace, ranging from 40 to 50 beats per minute, creating a tranquil and dreamlike atmosphere, as if gently guiding listeners into the mysterious world of ancient Egyptian civilization. However, the overall tempo range is broader, extending from 10 to 130 beats per minute (Figure 1). This strikingly wide tempo range demonstrates the work’s exceptional temporal plasticity in performance.

Beyond shared characteristics, diverse patterns of different schools emerge in the macro-tempo choices. In terms of the average tempo ranking, Western European pianists adopt the fastest pace, followed by Eastern European performers, while Chinese pianists tend to choose a relatively slower tempo. General average speed ranking (from fastest to slowest): Western European pianists > Eastern European pianists > Chinese pianists.

Notably, Arrau, representing the German school, and Chen Sa, belonging to the Chinese school, are two examples that depart from their respective traditions, exhibiting the extremes of the tempo spectrum. Their recordings represent the most extreme explorations of the piece’s lower temporal threshold (marked with a purple box in Figure 1).

### Macroscopic tempo: shaping

Different patterns of tempo variations emerge in macro-tempo variations, conveying various concepts in the shaping of the temporal development of the entire prelude. The performances of French and Italian pianists exhibit distinctive temporal architectures.

**Figure 2:**
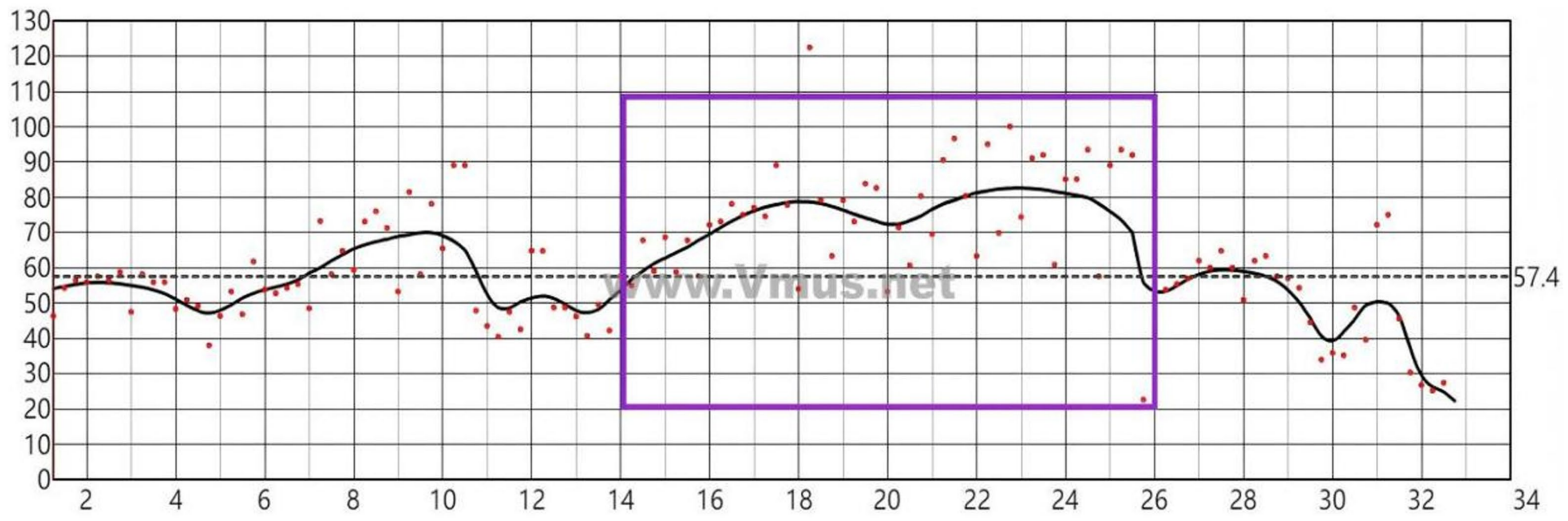
Maurizio Pollini Macro Tempo Curve.

**Figure 3:**
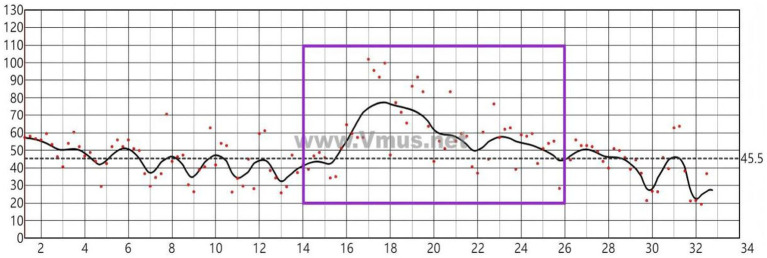
Jean-Yves Thibaudet Macro Tempo Curve.

In the mid-section climactic passage from measures 14 to 26, their interpretations feature coherent and clear tempo transitions: first accelerating forward, then decelerating in retreat, forming an arched tempo peak that represents a powerful climax. Maurizio Pollini (Figure 2), Jean-Yves (Figure 3), and Thibaudet are typical representatives of such a temporal approach. Their performances reinforce the work’s structural arch and highlight the narrative progression in temporal designs.

**Figure 4:**
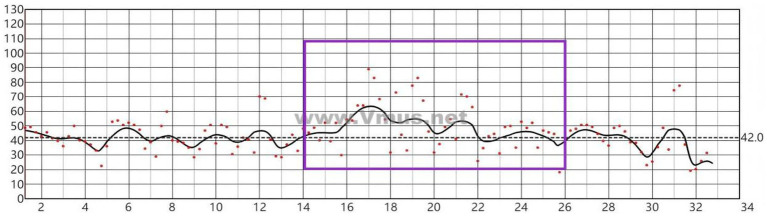
Sviatoslav Richter Macro Tempo Curve.

**Figure 5:**
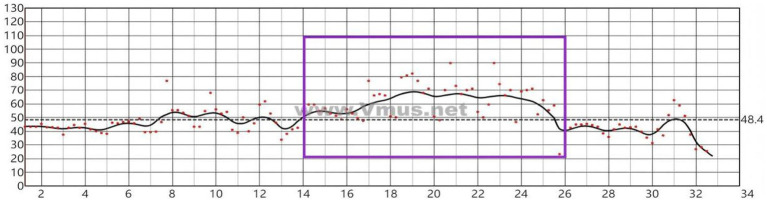
Friedrich Gulda Macro Tempo Curve.

In contrast, pianists from Eastern Europe and German-Austria present a distinct type of temporal architecture. Although they also establish an arched tempo peak in the climactic segment, their approach is more gradual and subdued. The progression towards the climax is more nuanced, leading to a broader and less prominent peak. This indicates a higher degree of overall temporal stability, with a preference for a more consistent pulse and a more profound, perhaps more introspective interpretation. Representative instances encompass Sviatoslav Richter (Figure 4) and Friedrich Gulda (Figure 5).

**Figure 6:**
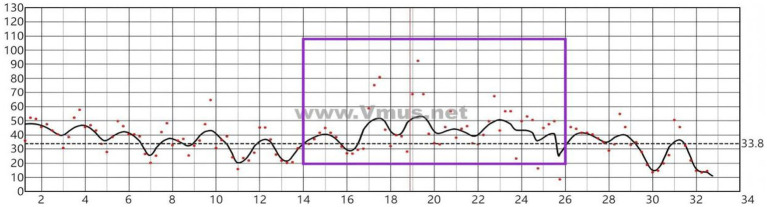
Chen Sa Macro Tempo Curve.

**Figure 7:**
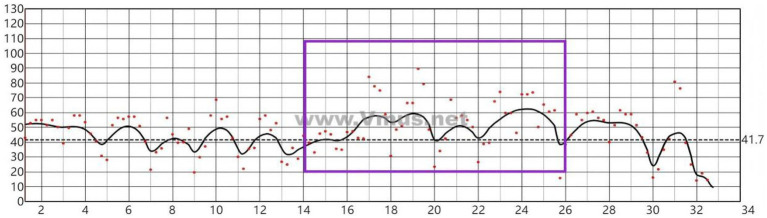
Yin Chengzong Macro Tempo Curve.

The interpretive methodology adopted by Chinese pianists offers a remarkable alternative. Eschewing a distinct climactic arc, performers like Chen Sa (Figure 6) and Yin Chengzong (Figure 7) utilize frequent and conspicuous tempo variations and pauses. This gives rise to a “free rhythm” which endows the music with an organic and unpredictable characteristic. Through this interpretive approach, the pianists enhance the music’s subtlety and evoke its envisioned spatial dimensions. Their performances emphasize poetic allusion and fluid expression over structural drama. The silences generated by dramatic pauses and decelerations can be regarded as *liubai (留白)*, or “artistic void,” a concept deeply rooted in Daoist “wu” (*无*, non-being). In traditional Chinese aesthetics, liubai serves as intentional negative space, fostering aesthetic resonance through potentiality, dynamic equilibrium, and imaginative involvement.

**Figure 8:**
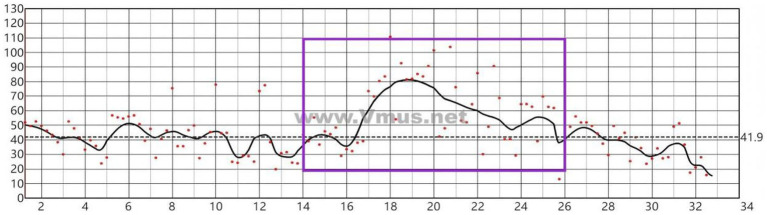
Fou Ts’ong Macro Tempo Curve.

Transcending national and regional patterns, Fou Ts'ong (Figure 8) emerges as a distinctive synthesizer of European and Chinese traditions. His performance uniquely combines the frequent tempo fluctuations of an Eastern “free rhythm” with the clear structural logic of a Western climactic arch. This fusion enables dynamic, fluid expression within a firmly coherent narrative framework, reflecting a profound personal integration of Eastern and Western performance aesthetics. As a Chinese pianist who moved to and studied in Europe, Fou Ts'ong preserves the sensibilities of traditional Chinese art while striving to integrate the aesthetic essences of both East and West. As foreign media once remarked of his artistry: “Fou Ts'ong’s performance art is born from the high degree of clarity inherent in the Chinese artistic tradition.”

### Micro-rhythmic rubato: theme “approaching”

**Figure 9:**
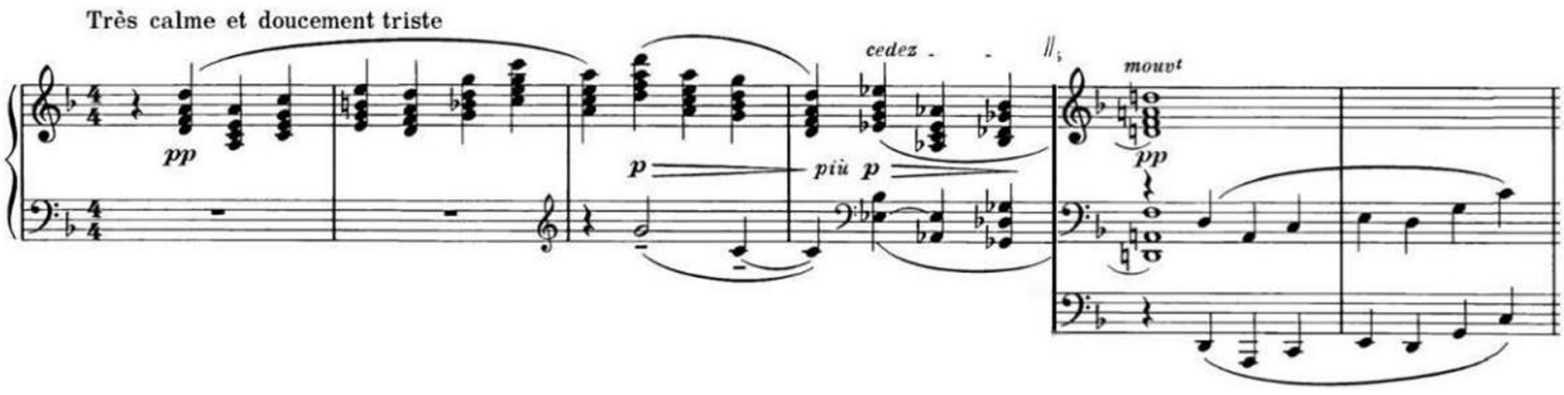
mm.1—7, Theme “Approaching”.

The opening theme, “Approaching” (Figure 9), represents the initial focal point for our micro–rhythmic analysis. Measures 1 to 7 are composed of stable quarter–note block chords. The four-phrase unit adheres to a distinct long-short-short-long pattern. This structural composition, in conjunction with a parallel voice texture, establishes a fundamental sense of rhythmic order. However, this order is inherently susceptible to subtle variations, a characteristic that performers instinctively leverage.

Within the “Approaching” theme, tempo variations are most conspicuous at the breathing intervals between musical phrases, while the remainder of each phrase remains comparatively stable. On this basis, three phrasing models have emerged across different interpretations. Notably, none of these interpretations aligns with the provisional clusters or with any established national or regional artistic traditions. Instead, the interpretations presented here primarily reflect the interplay of similarities and differences among individual artistic perspectives, shaped not by collective tradition, but by personal expressive choices.

**Figure 10:**
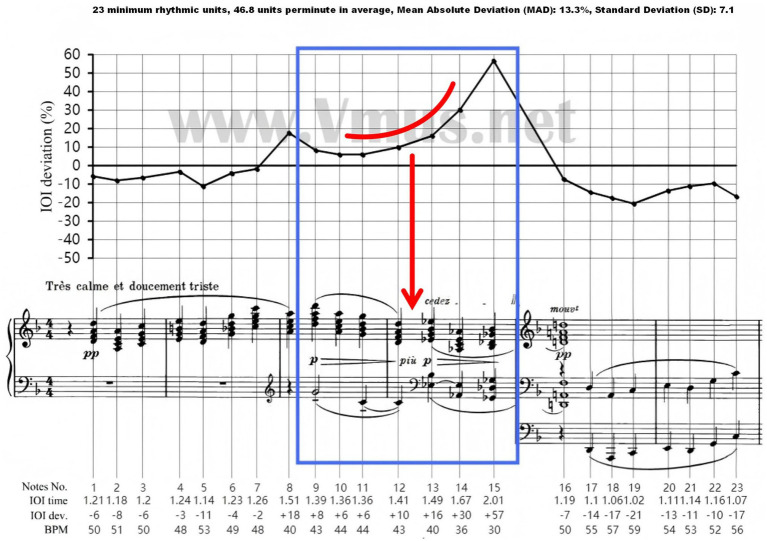
Walter Gieseking “Approaching” theme IOI deviation curve.

The first pattern entails the amalgamation of the second and third phrases through their execution within a single breath, without pausing to indicate the comma between the phrases. This results in the transformation of the overall phrasing pattern into a structure composed of three long phrases. Performers who adhere to this pattern include Walter Gieseking, Yin Chengzong, and Sviatoslav Richter, with Walter Gieseking serving as an exemplar (Figure 10). Such treatment reconfigures the original phrasing, thereby enhancing the hierarchical structure and developmental logic of the musical material within the local framework.

**Figure 11:**
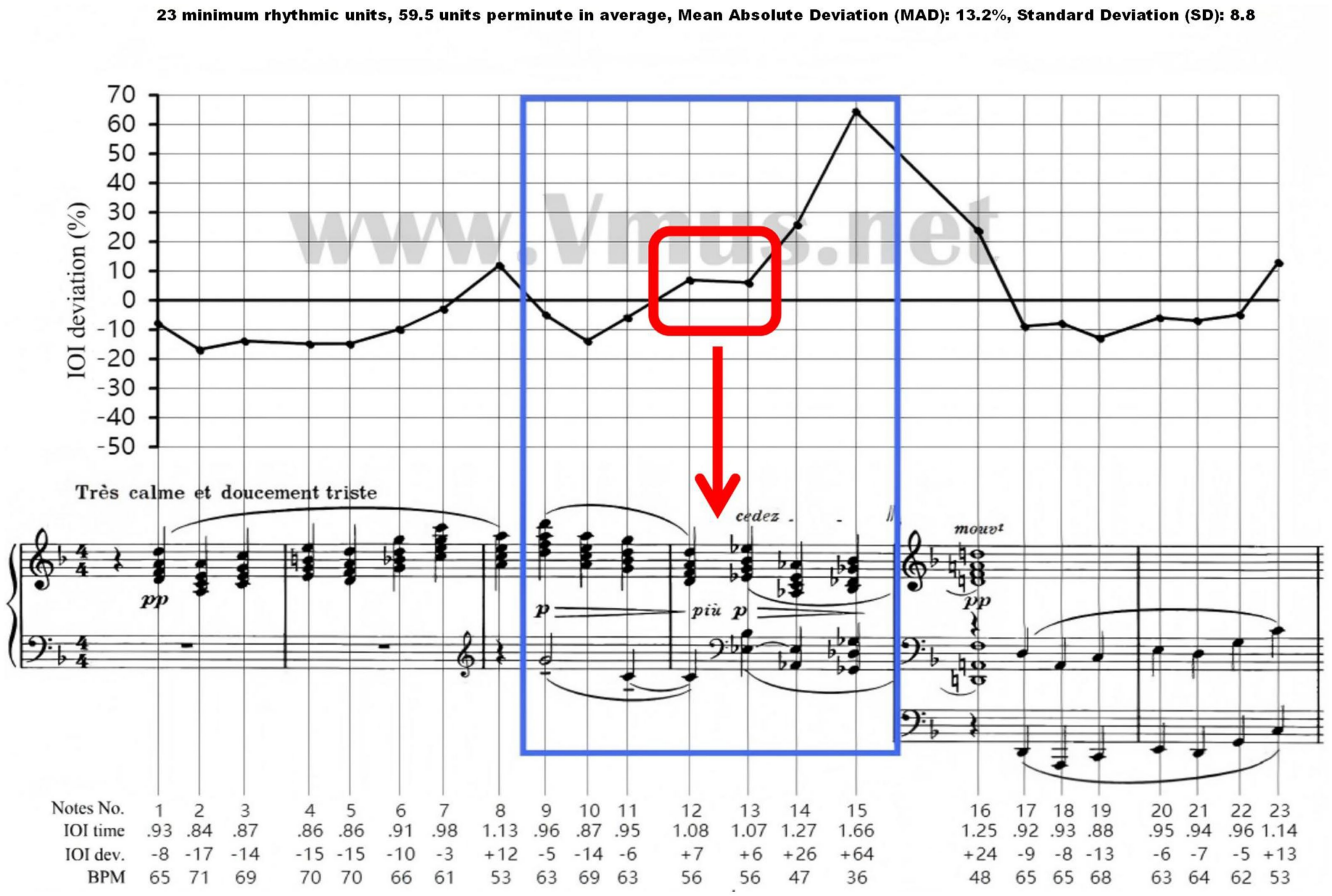
Pascal Rogé “Approaching” theme IOI deviation curve.

The second pattern is characterized by a relatively short breathing interval between the second and third musical phrases. Musicians who adopt this approach include Youri Egorov, Maurizio Pollini, Chen Sa, Jean-Yves Thibaudet, and Pascal Rogé. Here, Pascal Rogé (Figure 11) is presented as an example. The breathing interval between the second and third phrases is shorter than that between the first and third phrases. This arrangement also strengthens the connection between the second and third phrases while essentially conforming to the notated phrasing demarcations.

**Figure 12:**
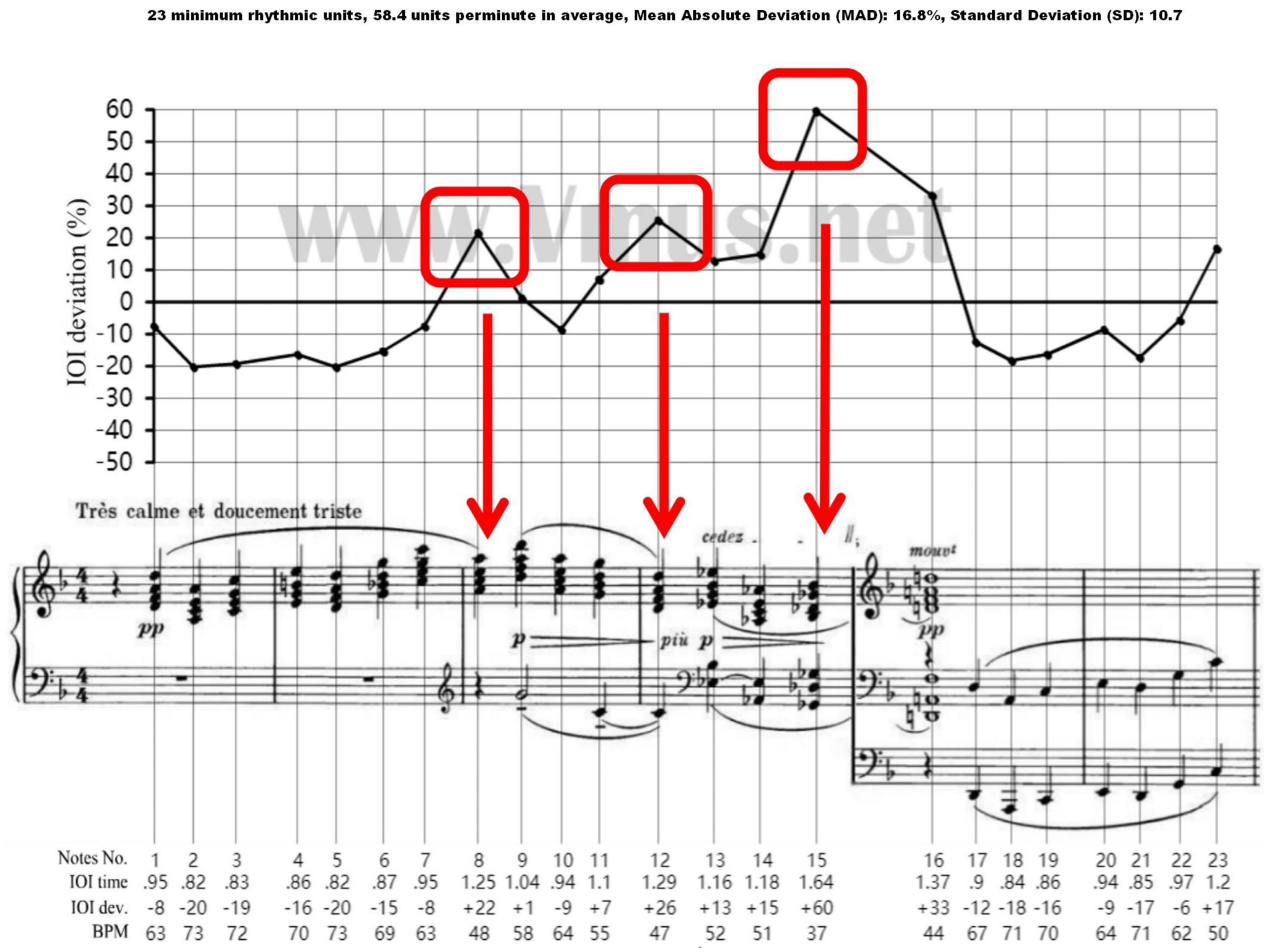
Arturo Benedetti Michelangeli “Approaching” theme IOI deviation curve.

The third pattern is characterized by distinct breathing intervals among all four phrases, strictly conforming to the long-short-short-long pattern specified in the score. Pianists who have employed this approach encompass Arturo Benedetti Michelangeli, Laurent Aimard, Fou Ts'ong, Krystian Zimerman, and Claudio Arrau, with Michelangeli (Figure 12) serving as a representative case.

### Micro-rhythmic rubato: theme “Viewing one of the jars”

**Figure 13:**
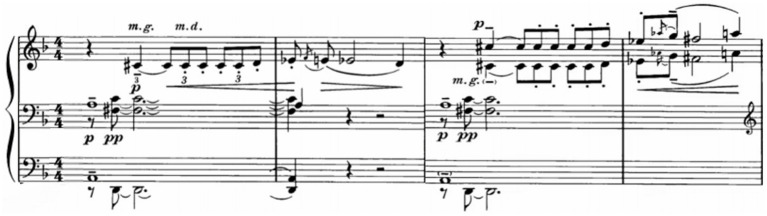
mm.7—11, Theme “Viewing one of the jars”.

Encompassing Measures 7–11 (Figure 13), the theme of “viewing one of the jars” initiates the larger middle section, namely “viewing the 4 jars.” This section is composed of two fundamentally repetitive phrases. The melodic line, initially presented as individual notes, is strengthened through octave doubling when it is repeated. The rhythmic characteristic of this passage is delineated by a distinct dramatic temporal arc: it commences in a state of stillness, gradually intensifies in activity, and subsequently resolves back into tranquility, thus constructing a miniature narrative of observation.

**Figure 14:**
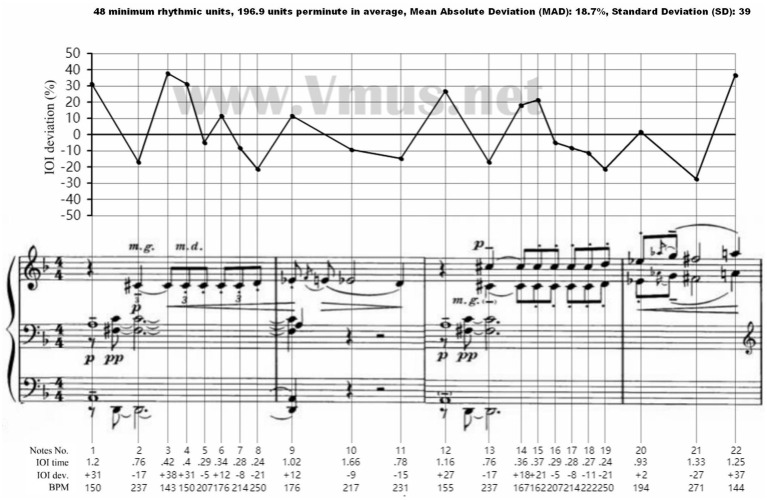
Maurizio Pollini “Viewing one of the jars” theme IOI deviation curve.

Within this thematic context, the performance styles of French, Italian, and German-Austrian musical traditions demonstrate a comparable approach. These styles are characterized by frequent localized rubato nuances, with the IOI curve presenting minor, serrated fluctuations. This approach not only conveys an air of improvisational subtlety but also sustains a regulated equilibrium within the confines of restrained rubato. Maurizio Pollini’s performance (Figure 14) serves as an exemplary manifestation of this style.

**Figure 15:**
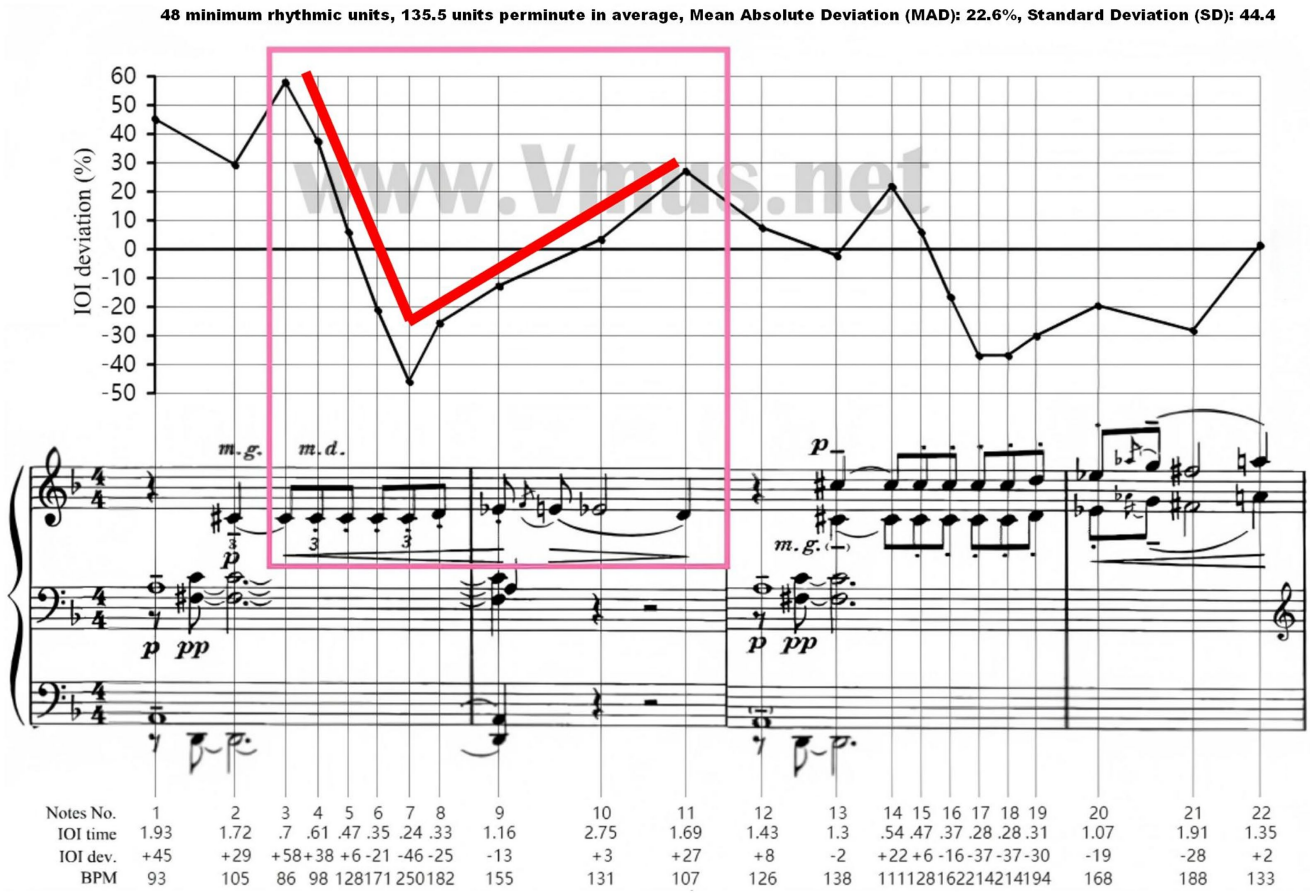
Krystian Zimerman “Viewing one of the jars” theme IOI deviation curve.

In the performances of Eastern European and Chinese pianists, a progressive rubato tension manifested, giving rise to a long arc of push–pull dynamics. Specifically, within the Eastern European performance style, a progressive acceleration and deceleration transpire from the triplets to the end of the phrase, ultimately shaping a V-shaped curve. This distinctive tempo manipulation maintains melodic tension and substantially elevates the dramatic expressiveness of the music. Krystian Zimerman’s performance (Figure 15) stands as a typical exemplar of this model.

In contrast, Chinese performers articulate a more intricate rhythmic narrative. They typically commence an acceleration at the triplets, followed by a gradual deceleration into the subsequent measure. This is immediately succeeded by a second, discernible acceleration towards the conclusion of the phrase. The outcome is a layered, push-and-pull effect that accentuates the melodic cadence with a distinct sense of rhythmic undulation.

**Figure 16:**
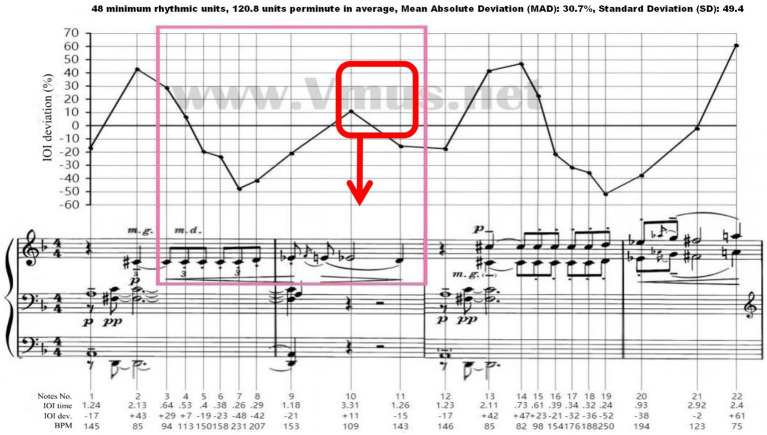
Fou Ts'ong “Viewing one of the jars” theme IOI deviation curve.

Upon closer scrutiny, there are even more subtle differences among individual artists. In the interpretations by Fou Ts'ong (as exemplified in Figure 16) and Yin Chengzong, the acceleration commences from the penultimate half-note and persists until the end of the phrase, imparting momentum to the subsequent phrase. This condenses the transitional space between phrases while creating a temporal juxtaposition against the subsequent deceleration.

**Figure 17:**
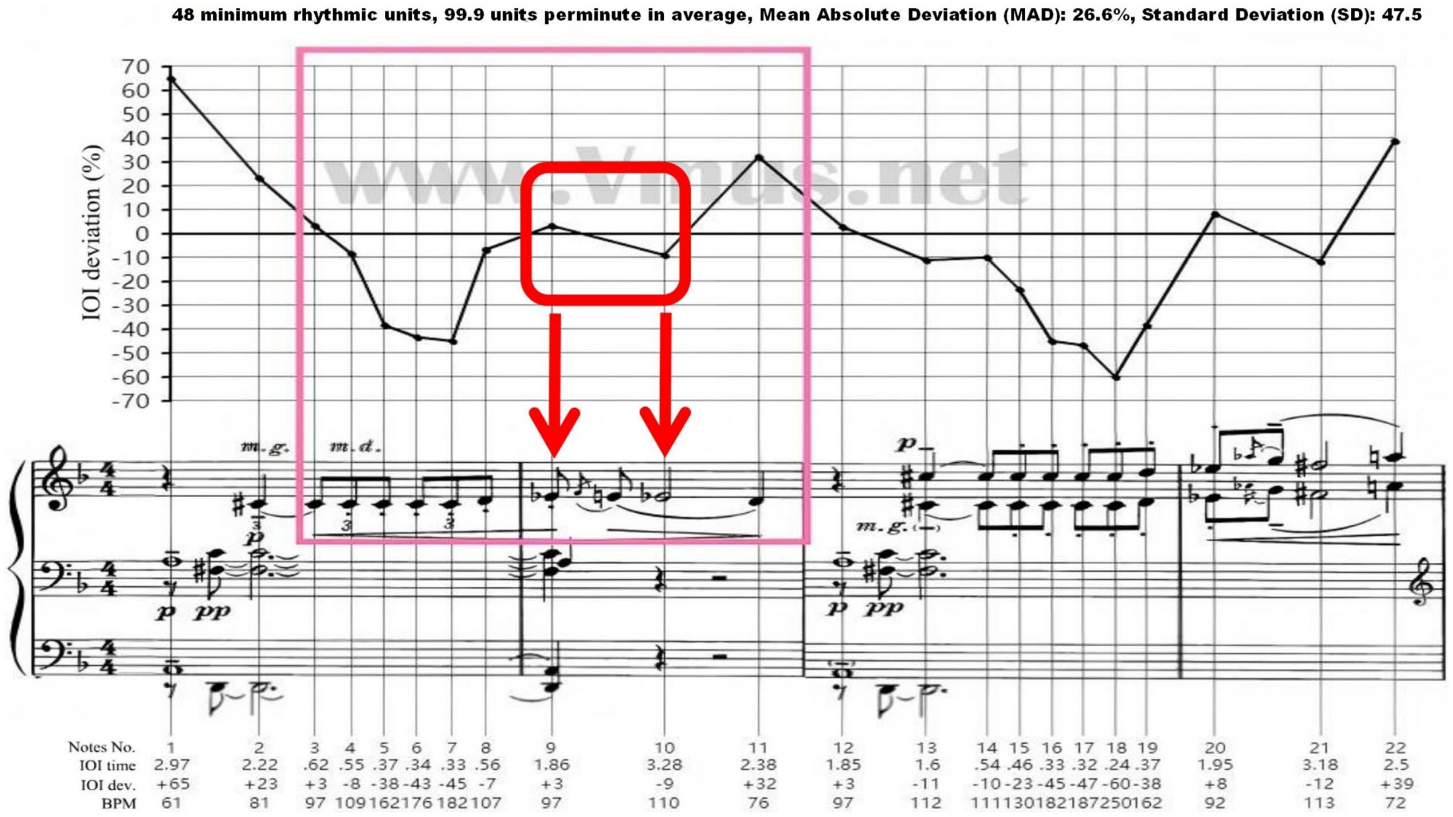
Chen Sa “Viewing one of the jars” theme IOI deviation curve.

In Chen Sa’s performance (Figure 17), a renewed acceleration initiates from the first eighth–note following the triplets, followed by a deceleration starting from the penultimate half–note, which continues steadily until the phrase concludes. This modulates the phrase’s resolution into a relaxed dissolution after the accelerando’s impetus, prolonging the transitional space while initiating the entrance of the subsequent phrase with a more ethereal ambiance.

**Figure 18:**
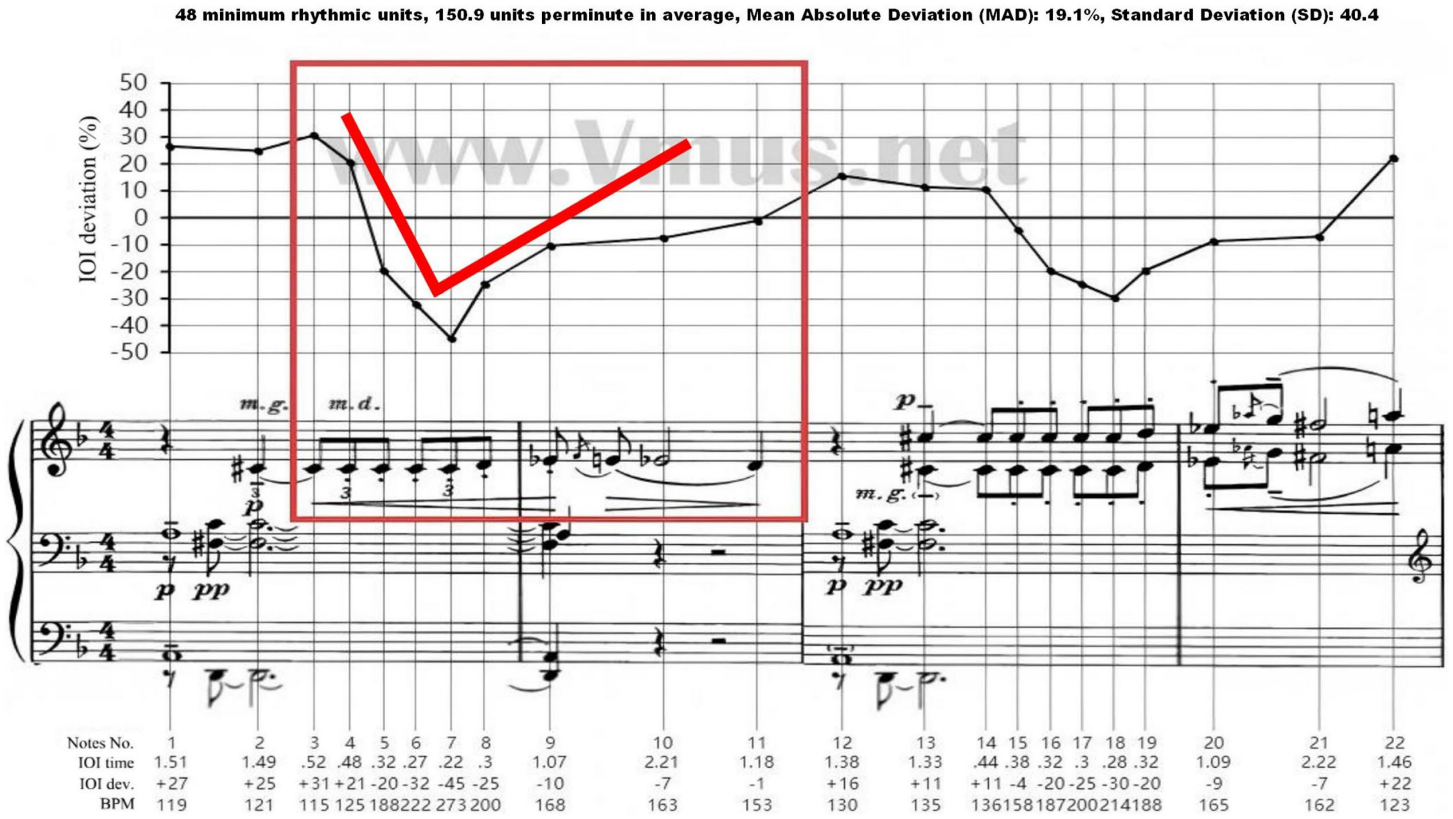
Friedrich Gulda “Viewing one of the jars” theme IOI deviation curve.

Friedrich Gulda (Figure 18) represents a unique instance of stylistic crossover. As a performer deeply entrenched in the German-Austrian tradition, he deviated from the more conventional German school approach characterized by minor and controlled local rubato nuances. Instead, he embraced a progressive V-shaped rubato in line with the practices of the Eastern European pianists. By doing this, he surpasses the limitations of a single interpretive strategy. This cross-stylistic phenomenon exemplifies the tendency toward dynamic integration and exchange among diverse artistic traditions.

## Conclusion

This study offers three key findings:

First, temporal elasticity serves as a vehicle for the creative reconstruction of the score.

Performances of *Canope* involve creative reconstruction of the score through temporal elasticity. Flexible phrasing reorganizes musical material through variations in “breathing” between phrases. The “Approaching” theme, therefore, reveals how structural order and individual expression coexist: the score provides a skeleton of rhythmic stability, but it is the performer’s subtle pause and stretch that brings the music to life as a uniquely personal utterance.

Second, macro-temporal shaping and micro-rhythmic rubato patterns serve as stylistic lineage-associated tendencies.

Within the provisional artistic-tradition clusters, specific temporal treatment patterns suggest potential associations with established artistic lineages:

1. Western European traditions (French/Italian/German-Austrian): Frequent, subtle tempo fluctuations within localized rubato, balancing fluid spontaneity with precise temporal control.
2. Eastern European traditions (Russian/Polish): Gradual rubato trajectories that sustain melodic tension and heighten dramatic expression.
3. Chinese tradition: Unexpected rhythmic turns and deliberate pauses woven into gradual temporal development, conjuring up associations with the traditional Chinese aesthetic concept *liubai* (artistic void) and creating a poetic and fluid expression.

Crucially, these observed patterns remain specific to the analyzed repertoire and parameters, and should not be interpreted as deterministic features of respective artistic traditions. Actual performance outcomes are significantly influenced by a host of confounding variables, including individual artistic agency, cross-cultural experiences, and evolving historical contexts.

Third, deviations serve as sites of innovation and cross-lineage dialogue.

Performances that diverge from the observed tendencies within the provisional clusters are shown to enact individual artistic agency that transcends conventional stylistic boundaries, thereby serving as sites of innovation and cross-lineage dialogue.

This study endeavors to construct a temporally centered analytical framework for the study of musical performance. Its scholarly contribution lies in situating strategies of temporal manipulation within the dual dimensions of performance cultural context and individual agency. Through a comparative examination of temporal parameter variations across different recorded interpretations of Debussy’s *Canope*, the research preliminarily uncovers potential associative patterns between performance styles and specific artistic traditions. It must be noted, however, that these observed patterns represent tentative findings derived from a particular sample set, and any claims regarding their explanatory power and general applicability should be advanced with due caution.

The observed correlations between performance styles and cultural backgrounds may be influenced by multiple confounding variables, including but not limited to: pedagogical lineages of performers, technological constraints and aesthetic conventions of recording eras, production requirements imposed by record companies, and individual artistic preferences of interpreters. Consequently, attributing these associations directly to the transmission of specific artistic traditions demands heightened circumspection. Establishing more robust causal linkages would require integration of oral history documentation, pedagogical archives, and longitudinal intergenerational comparative studies.

## Data Availability

The original contributions presented in the study are included in the article/supplementary material, further inquiries can be directed to the corresponding author.
